# A horse and a zebra: an atypical clinical picture including Guillain-Barré syndrome, recurrent fever and mesenteric lymphadenopathy caused by two concomitant infections

**DOI:** 10.1007/s15010-020-01397-5

**Published:** 2020-03-03

**Authors:** Felix Amereller, Christian Lottspeich, Grete Buchholz, Karl Dichtl

**Affiliations:** 1 Medizinische Klinik und Poliklinik IV, Klinikum der Universität München, Ludwig-Maximilians-Universität München, Munich, Germany; 2 Department of Neurology, University Hospital, Ludwig Maximilian University of Munich, Munich, Germany; 3 Max von Pettenkofer-Institut für Hygiene und Medizinische Mikrobiologie, Medizinische Fakultät, LMU München, Munich, Germany

**Keywords:** *Campylobacter jejuni*, *Yersinia pseudotuberculosis*, Guillain-Barré syndrome, Mesenteric lymphadenopathy, Mesenteric lymphadenitis, Pseudoappendicitis

## Abstract

**Background:**

While *Campylobacter jejuni* represents the most common cause of bacterial gastroenteritis, *Yersinia pseudotuberculosis* infections are very rarely diagnosed in adults.

**Case:**

We report on a previously healthy patient who presented several times at our hospital with fever, Guillain-Barré syndrome, recurrent abdominal symptoms and distinct mesenteric lymphadenopathy, respectively. This complicated and diagnostically challenging course of disease was caused by a *C. jejuni* and *Y. pseudotuberculosis* coinfection. Antibiotic treatment with doxycycline was effective.

**Conclusion:**

Broad serology testing was crucial to discover that two concomitant infections were causing the symptoms. This case demonstrates that when a clinical picture is not fully explained by one known infection, another infection with the same underlying risk factor has to be considered, hence “a horse and a zebra”.

**Electronic supplementary material:**

The online version of this article (10.1007/s15010-020-01397-5) contains supplementary material, which is available to authorized users.

## Case report

A 28-year-old Caucasian male presented with fever and myalgia at our emergency department (= day 1). The patient reported having been on vacation on La Réunion until nine days prior, where a medium-level dengue epidemic had been declared by the WHO. His past medical history was uneventful; he was not on any medication. Physical examination revealed a body temperature of 39.7 °C but was otherwise unremarkable. Routine laboratory investigations were without pathological findings except for a C-reactive protein (CRP) of 2.5 mg/dl. Tests for *Dengue virus* and *Chikungunya virus* were ordered. The patient received metamizole and was discharged with a daily follow-up schedule which, however, he did not comply with. The microbiological laboratory tests returned negative. After three days, fever subsided and was followed by watery diarrhoea which lasted for three more days. On day 7, the patient had fully recovered.

On day 8, the patient noticed a weakness in his lower limbs which continuously worsened overnight. Thus, he presented in our neurological clinic the next morning. Physical examination revealed flaccid tetraparesis with a level of strength of 4/5 (MRC scale). Muscle reflexes of the upper limbs and patellar reflexes were decreased, Achilles reflexes were absent bilaterally. There were no sensory deficits; position sense and vibration sense were intact. Cerebrospinal fluid analysis was unremarkable. Electroneurography disclosed reduced amplitudes of compound muscle action potentials in tibial, peroneal and ulnar nerves. Half of the examined nerves displayed increased distal motor latency and total loss of F-waves, while sensory nerve action potentials were normal all over. Hence, pure motor axonal demyelinating polyneuropathy with acute onset, consistent with Guillain-Barré syndrome (GBS) was diagnosed. Furthermore, western blot for serum anti-ganglioside antibodies was highly positive for anti-GM2 IgM antibodies and borderline positive for anti-GM1 IgM antibodies, thus supporting the diagnosis of GBS. The occurrence of GBS raised the suspicion of a recent *Campylobacter jejuni* infection which was serologically confirmed (*Mycoplasma pneumonia*, another common trigger of GBS, and *Zika virus* infection were excluded).

As the clinical condition deteriorated rapidly with inability to walk occurring within the first 48 h, treatment with intravenous immunoglobulins was initiated (total dose 140 g over 5 days). Clinical nadir was reached after three days and the patient regained independent walking within the first week. He was transferred to a neurorehabilitation institution where he was treated for three weeks. Except for a temporary elevation of transaminases (ALT 661 U/l, AST 126 U/l) and a distortion of the left knee due to several falls, further recovery proceeded without complications.

In week 10, the patient presented at our clinic again. He complained of fever, loss of appetite, abdominal bloating, constipation and a dull pain in the right lower abdomen. He reported suffering from these symptoms periodically; they had first occurred three weeks ago and lasted for a couple of days, then completely disappeared and reoccurred six days ago. The onset as well as the disappearance of these symptoms was sudden. On enquiry, the patient could not think of any potential triggers preceding these episodes. Diarrhoea had not occurred.

**Fig. 1:**
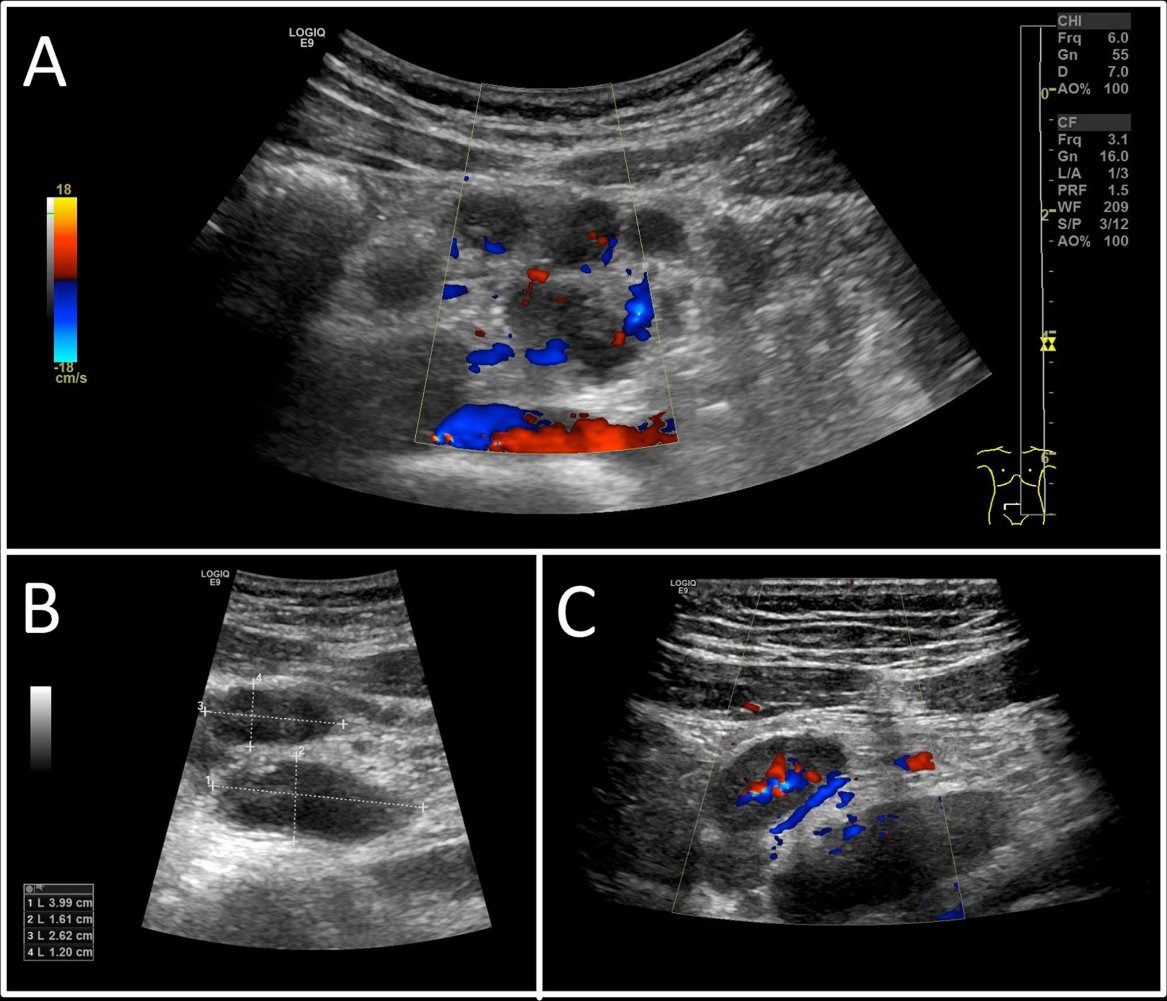
Abdominal ultrasound examination reveals multiple enlarged mesenteric lymph nodes in the right lower quadrant. **a** Hypoechoic lymph nodes surrounded by hyperechoic mesenteric tissue. **b** Diameters of the two largest oval shaped lymph nodes (max. diameter 4 cm). **c** Hilus perfusion of the lymph nodes depicted by duplex ultrasound

The patient’s vital signs were all normal except for a body temperature of 38.8 °C. Compared to his first presentation, he had lost 11 kg (15% of his previous body weight). The abdominal examination was pertinent for tenderness on palpation in the right lower quadrant. Broad laboratory investigations were unremarkable apart from an elevated CRP of 8.5 mg/dl and a slightly elevated LDH. Abdominal ultrasound revealed a distinct mesenteric lymphadenopathy with > 10 pathological lymph nodes (max. 4 × 1.6 cm) (Fig. 1). The largest mesenteric lymph nodes were found in the right lower quadrant and were painful when pressed with the ultrasound probe. Retroperitoneal, inguinal, supraclavicular, axillary and cervical lymph nodes as well as the appendix and colon appeared normal on ultrasound examination.

A broad microbiological testing was initiated. While stool cultures remained negative, serology was positive for *Y. pseudotuberculosis* (Table 1). Antibiotic treatment was initially started with azithromycin (500 mg p.o., QD, for 3 days) and switched to doxycycline (100 mg p.o., BID, for 10 days) after receiving the serology results.

**Table 1:** Results of anti-*C. jejuni-* and anti-*Y. pseudotuberculosis*-antibody testing from sera sampled at three different time points during the course of disease

	*C. jejuni*				*Y. pseudotuberculosis*		
	Blot		ELISA		Blot		Widal test
	IgA	IgG	IgA	IgG	IgA	IgG	
Week 1 (occurrence of GBS)	Positive	Positive	Positive	Positive	Negative	Negative	Negative
Week 10 (lymph-adenopathy)	Negative	Positive	Positive ↓	Positive ↓	Positive	Positive	Borderline ↑
Week 12 (follow-up visit)	Negative	Positive	Negative	Negative	Positive	Positive	Borderline

For this retrospective comparison, parallel testing of all sera was performed. Upwards and downwards arrows indicate a significant (min. fourfold) increase and decrease in titers, respectively

On control ultrasound two weeks later (week 12), the lymphadenopathy was clearly regressive in all affected regions with the largest mesenteric lymphnode measuring 2 × 0.7 cm. In a telephone follow-up two months later, the patient reported no further episodes of fever or abdominal symptoms. Regarding GBS, he had not regained his full physical capacity but continued to improve constantly.

## Discussion

*Campylobacter jejuni* is a Gram-negative, rod-shaped bacterium that naturally colonizes the digestive track of both wildlife and domestic farm animals [1]. It usually infects humans through the ingestion of contaminated meat and poultry [2, 3]. Rarely, infections also occur through ingestion of contaminated water, uncooked milk or via person-to-person transmission [2]. Incubation time is commonly less than five days but can range up to ten days [4] with a lower infection dose possibly leading to longer incubation time [5]. As it was the case in our patient, campylobacteriosis often sets in with unspecific, flu-like prodromal symptoms lasting for 1 or 2 days. This is followed by acute enteritis with watery diarrhoea and sometimes crampy abdominal pain that lasts six days on average. The infection is usually self-limiting and does not require specific treatment, however, it can lead to severe immunological complications. Due to molecular mimicry between its lipooligosaccharides and host gangliosides, *C. jejuni* is the major trigger of GBS [6]. Here, it is associated with a pure motor syndrome, as it occurred in our patient, and a slower recovery [7]. *C. jejuni* is the most common cause of bacterial gastroenteritis in developed countries (e.g., estimated incidence in Germany: 53-81/100,000 persons/year [8]). The prevalence in developing countries is even higher, making it the most frequent travel associated enteric infection [9].

*Yersinia pseudotuberculosis* on the other hand is a very rare cause of enteric disease. The number of diagnosed cases varies strongly in different studies (Finland: 0.6–0.8/100,000 persons/year [10]; USA: 0.04/1,000,000 persons [11]; Germany: 0.03/100,000 persons/year [12]). *Y. pseudotuberculosis* is a Gram-negative, exotoxin producing rod [13]. It can be found ubiquitously in various animals including birds, game animals and pigs [14]. Outbreaks have been linked to the ingestion of fresh produce as well [10, 14]. This is certainly favoured by the species’ resilience to extremes of temperature and minimal nutritional requirements [13]. In humans, the infection leads to different symptoms depending on the patient’s age and the geographical origin of the strain. Symptoms of infection caused by strains predominant in Europe are fever, acute terminal ileitis (pseudoappendicitis) and mesenteric lymphadenitis. Notably, these symptoms mostly occur in children and young adults [12]. Due to different virulence factors, endemic strains in far-eastern Russia and Asia also cause scarlatiniform rash, desquamation and erythema nodosum [15]. Incubation time is commonly 5–10 days but can be as long as 21 days [16].

The incubation time in our patient seemed to differ, making this cases chronological sequence remarkable. It is extremely unlikely that these two infections, one of them very rare, occurred within few weeks in an immunocompetent person without any correlation between each other. However, poor food hygiene is the common main risk factor for both infections. Moreover, pig herds have been shown to carry both *Campylobacter* spp. and *Yersinia* spp.[17]. Therefore, we considered a simultaneous infection to be most probable. Assuming this, the incubation time until the first symptoms presumably associated with *Y. pseudotuberculosis* occurred would have been at least seven weeks. This seems astonishingly long, however, during an outbreak of *Y. pseudotuberculosis* length of illnesses up to six months were observed with symptoms occurring later in adults than in children [18]. This is probably due to the species’ ability to colonize and persist in intestinal and lymph tissues with the aid of specific outer membrane proteins [19].

In addition, antibody titres to *Y.* *pseudotuberculosis* were shown to rise particularly late in many patients [18, 20]. This matches the serological findings in our case well. Neither *C. jejuni* nor *Y.* *pseudotuberculosis* was isolated from stool cultures, probably because the patient did not present while having acute diarrhoea. Typically, by the time GBS occurs serology is more sensitive for diagnosing a recent *C. jejuni* infection than stool cultures [5].

The overlap of two concomitant infectious diseases resulting in a complex clinical picture not attributable to any specific entity made this case a diagnostic challenge. Following the principal that “when hearing hoofbeats, one should think of a horse not a zebra”, the obvious suspicion was that a persisting campylobacteriosis caused the recurrent abdominal symptoms and fever. Nevertheless, other symptoms like the extensive lymphadenopathy did not seem to fit in. Retrospectively, however, it became clear that two different infections had presented in an absolutely exemplary way. This is particularly remarkable for the *Y.* *pseudotuberculosis *infection whose full clinical picture is very rarely found in adults. This case demonstrates that in a patient with one known infection, another infection with the same underlying risk factor should always be taken into account. Therefore, sometimes both “a horse and a zebra” have to be considered.

## Electronic supplementary material

Below is the link to the electronic supplementary material.

Supplementary file1 (TIF 17794 kb)
