# Supplementary figures and images for: A horse and a zebra: an atypical clinical picture including Guillain-Barré syndrome, recurrent fever and mesenteric lymphadenopathy caused by two concomitant infections
Source: Infection. 2020 Mar 3;48(3):471–5. doi: 10.1007/s15010-020-01397-5 (PMC7256024; doi:10.1007/s15010-020-01397-5)

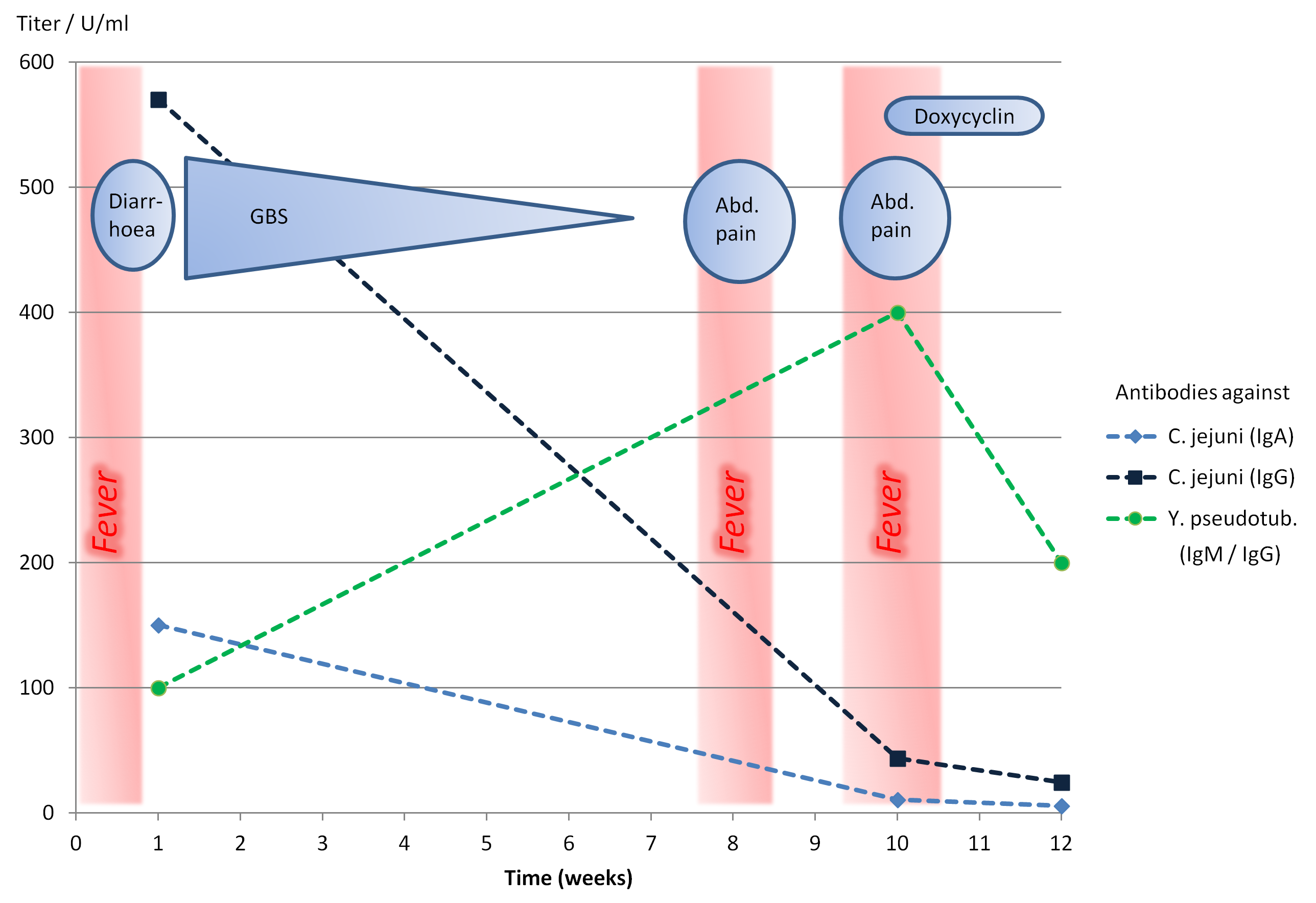

Supplement: Supplementary file 1 — Supplementary file1 (TIF 17794 kb) [file 15010_2020_1397_MOESM1_ESM.tif]
